# Earliest Mule Remains from Early Bronze Age Central Anatolia

**DOI:** 10.3390/ani14101397

**Published:** 2024-05-07

**Authors:** Can Yümni Gündem

**Affiliations:** Faculty of Arts and Sciences, Head of the Prehistoric Archaeology, Batman University, Batı Raman Campus/Room: 76, 72060 Batman, Türkiye; canyumni.gundem@batman.edu.tr

**Keywords:** donkey, mule, Central Anatolia, Early Bronze Age, Assyrian Trade Colonies Ages, zooarchaeology, archaeozoology

## Abstract

**Simple Summary:**

The Assyrian Trade Colonies Age, from the EBA to MBA, marked a shift in the trading system between Anatolia and Mesopotamia due to increased mineral trade. Assyrian traders transported tin, textiles, and valuable stones and metals, paying taxes to local rulers. They established settlements and established *Karum* colonies in major cities and *Wabartum* stations in smaller ones. It is known that donkeys and mules were used as caravan animals towards the end of the Early Bronze Age in Central Anatolia. However, we have not identified enough archaeological material to prove the existence of mules in particular. Animal bone remains recovered from the Derekutuğun mining settlement were examined, and especially the teeth of equids were further examined by the researcher. This study mentions the existence of the oldest known possible mules, especially based on the dental remains of equids found in Derekutuğun.

**Abstract:**

This paper discusses the discoveries of early donkey and the earliest mule remains in Central Anatolia from the site Derekutuğun. This site represents the remains of a village dating back to the Early Bronze Age and Assyrian Trade Colonies period, associated with mining. The archaeofaunal assemblage was studied by the author and his team using classical archaeozoological methods. The dental remains of the Equidae found at Derekutuğun have been re-examined and are described in this article. The dental evidence indicates that donkeys, and possibly the earliest mules ever found in Central Anatolia, were kept at this site. Although the paper is based on the archaeozoological remains, written sources from the period also support the faunal identification. Derekutuğun was a small settlement that specialized in processing copper ore, and which was an important hub for a trade network because of its extensive mining and extraction operations.

## 1. Introduction

Horses, with their noble and imposing stance, have always attracted more attention than donkeys and mules throughout history. However, it should not be forgotten that donkeys and mules have also played a crucial role in the development of human history by carrying heavy loads.

Donkeys and mules have played important roles in the history of mankind, especially in trade and communication between societies. For example, based on ancient texts, donkey caravans are known to have facilitated economic interactions between northern Mesopotamia and Central Anatolia in the early second millennium BCE. However, despite their importance, there are very few known remains of these livestock animals from early periods in Anatolia. As a result, the history of these important equids and their roles in developing complex economies of the Bronze Age world are poorly understood. This study presents the evidence of the early presence of donkeys and mules in Central Anatolia, with a focus on the remains discovered at the site of Derekutuğun, a mining settlement in the north of Central Anatolia (Çorum/Türkiye) dating primarily to the Early Bronze Age.

### 1.1. An Overview of the Archaeology of the Derekutuğun Settlement*1*

**Figure 1:**
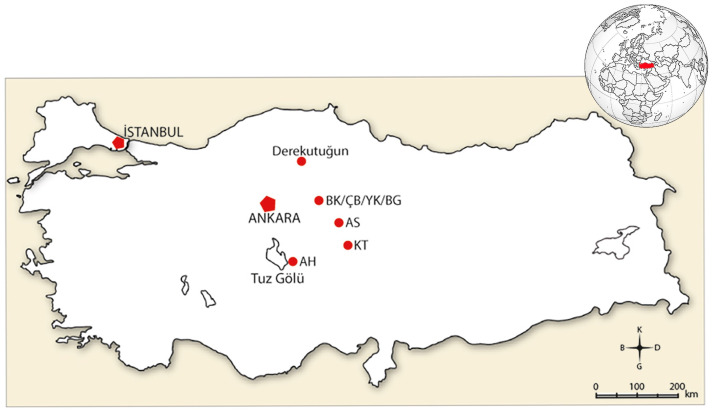
Map of Türkiye and the location of Derekutuğun and other mentioned important sites in the study (BK = Büyükkale, ÇB = Çamlıbel Tarlası, YK = Yarıkkaya, BG = Boğazköy, AS = Alişar, KT = Kültepe, AH = Acem Höyük). (Empty map from http://cografyaharita.com/haritalarim/4jdilsiz_turkiye_haritasi_s2.png, accessed on 30 April 2024).

The modern Derekutuğun Village is named after the Derekutuğun Mine Management. It is located in the western part of Bayat District in Çorum Province, approximately 20 km north of Kızılırmak River, at the southern end of the Köroğlu Mountains. The region is characterized by hilly and rugged terrain [1] (Figure 1). The archaeological site of Derekutuğun is located to the east of the modern village from which it derives its name with the site being divided into two main localities. Archaeological excavations took place in these two areas, including Erikli Mevkii, which provides settlement and chronological information, and Mazıöü Mevkii, an area where mining galleries are located. The results of surface surveys have revealed the presence of abundant copper deposits in the region [2]. It is believed that the copper extracted from Derekutuğun was used as a raw material in the Bronze Age due to its high purity [1].

A team of scientists from Heidelberg University conducted a brief surface survey in Derekutuğun and its vicinity in 1980. However, no archaeological artifacts related to ancient mining were discovered until more recent research at the site. However, the surface survey conducted in 2008 by Ünsal Yalçın and his team discovered traces of an archaeological site [1]. The initial excavations in the settlement took place between 2009 and 2012 under the scientific guidance of Ünsal Yalçın [3]. Excavations were conducted between 2015 and 2017, funded by the German Research Foundation (DFG, Deutsche Forschungsgemeinschaft). The Erikli Mevkii locality was excavated during these periods [4].

The site’s chronology was established based on the excavated ceramic sherds and small finds [1]. The pottery found at the settlement dates the first layers of the Derekutuğun settlement to the Chalcolithic Age. The studies conducted in Derekutuğun Erikli locality provide the settlement chronology of the mound, which from top to bottom includes the Roman Period, Hellenistic Period, Iron Age, Hiatus, Karum Period (Age of Assyrian Trade Colonies), Transitional Period from Early Bronze Age III (EBA III) to Middle Bronze Age III (MBA III), EBA III, EBA II, EBA I, and Chalcolithic Period [4] (Table 1). The archaeofaunal remains studied from the Early Bronze Age (EBA) Periods show oxidation as a result of the processing of metallic copper ore at the site, which gives the material a greenish color due to oxidation.

The Erikli Mevkii settlement area is located 300 m south of the Mazıönü locality, where the mining gallery is located. The density of miners’ stone tools and pottery fragments obtained during the survey suggested that Erikli Mevkii might be a settlement. Excavations in this locality began in 2015 and include four trenches [4] (p. 576).

Archaeologists discovered architectural remains from the Roman Period, Hellenistic Period, and Middle-Late Iron Age during the excavation in the Erikli I Trench. It has been observed that the walls of structures from the Late Iron Age are well preserved. The pottery obtained from the trench, especially the “s”-profiled bowls, also provides evidence for a Late Iron Age chronology. Pottery fragments from the Roman–Hellenistic Period were discovered during the excavations in the Erikli II Trench.

Excavations in the Erikli III Trench were conducted during the 2016 season, uncovering workshops, garbage pits, and stove areas beneath the Roman Period ruins. Mineral slag, blowtorches, and crucible pieces were found in the excavated areas, indicating mining activities. The garbage pits discovered beneath walls in the Erikli III trench are dated to the Hellenistic Period. Therefore, the Erikli III trench consists of two main layers: the Roman and Hellenistic Periods [4] (p. 577). The pottery finds discovered in the Erikli IV trench indicate the period from Early Bronze Age III to the Middle Bronze Age. Additionally, small excavated items such as spindle whorls and loom weights from the same trench are associated with settlements like Acemhöyük and Boğazköy [4] (p. 585). C-14 samples taken from the Erikli IV Trench produced dates ranging from 2200 to 1900 BC, supporting the existence of an occupation dating to the Assyrian Trade Colonies Age at Derekutuğu (pls. see bottom) [4] (p. 585). According to the results of C-14 analyses, the organic materials and pottery recovered from the mining galleries were dated as far back as 4000 BC to the Chalcolithic Period. However, mining activities at the Derekutuğun settlement began in the Early Bronze Age II [3] (p. 29) (Table 1).

### 1.2. The Assyrian Trade Colonies Ages in Anatolia

The Assyrian Trade Colonies Age is a unique archaeological period in Anatolia that spans the transition from the end of the Early Bronze Age (EBA) to the beginning of the Middle Bronze Age (MBA). During this period, the trading system based on obsidian between Anatolia and Mesopotamia, as well as Northern Syria, which had been established since the Neolithic Period, began to operate in the opposite direction due to the increased mineral trade. Assyrian traders transported tin and high-quality textiles requested by Anatolian cities using donkey caravans, and they exported valuable stones and metals, particularly gold, silver, and copper, which were produced and manufactured in Anatolia. In exchange for this trade, Assyrian traders also paid taxes to local rulers. The Assyrians established settlements in various parts of Anatolia to strengthen their trade network. Assyrian traders established Karum colonies in major cities and Wabartum stations in smaller cities. The largest known Karum is the Kaneš Karum, located at the site of Kültepe in Kayseri [5,6].

Assyrian trade in Anatolia extended from the Black Sea to Northern Syria and Mesopotamia, and from the Konya Plain in the west to beyond the Euphrates in the east. Assyrian trade colonies were established around 1950 BC and continued until around 1750 BC. During this trade, banking procedures, accounting practices, contract law, and commercial law were applied. The information about Assyrian trade in Anatolia is derived from ancient Assyrian cuneiform tablets. These tablets were the earliest written sources of Anatolia and were primarily excavated from the sites of Kültepe, Hattuša (Boğazköy), and Alişar. The sworn agreements were concluded by each indigenous ruler and the state of Assur. This agreement specified the rights and duties of indigenous rulers and Assur within a legal framework. Under this agreement, Assyrian trade continued without interruption for approximately 150 to 200 years. It was accepted as the first known documented “international trade”, which occurred between Anatolia and Assur (northern Iraq) [5,6].

## 2. Materials and Methods

The author and his students identified the animal remains from Derekutuğun at the Çorum Museum^2^. Most of the bone material studied originates from the transition layers of Early Bronze Age III to the Karum Period (EBA III/Karum), and the animal bone remains were primarily excavated from the interior of structures uncovered in the trenches in the Erikli Mevkii locality.

Animal bone remains from Derekutuğun were the subject of the master’s thesis submitted by S. SARI under the supervision of the author of this study. However, in her thesis, the remains of the equids were introduced under a single title and were not identified to species^3^. The author re-analyzed and re-evaluated especially the dental remains from equids, and the results are discussed in this paper.

The animal bone remains studied originated from excavations conducted under the direction of Prof. Dr. Ünsal YALÇIN in the years 2009–2011 and 2015–2017. A total of 4426 animal bone remains, weighing approximately 58.2 kg, from the three main periods of the Early Bronze Age were analyzed to understand the development of the animal-based subsistence economy. Traditional methods of archaeozoology were employed for the analysis of the animal bone remains. The archaeozoologist used visual methods to study animal remains, including bones and teeth. Each bone fragment was recorded in a Microsoft Excel spreadsheet, which included taxonomic and anatomical identification, degree of fragmentation, and the presence of cut marks, burning, and other modifications [7,8,9,10,11,12,13]. Additionally, the weight of each remaining bone was recorded to estimate their contributions to meat demand [14,15,16,17], and the age of the animals at the time of their death was determined using dental development and fusion of the epiphyses [9,17,18,19,20,21,22,23]. All measurable skeleton element remains from each species were measured and recorded during the identification of the animal bone assemblage [15,23,24,25,26,27,28].

In the archaeozoological studies conducted in this region and from similar archaeological periods, the most intensive kept livestock group typically consists of small ruminants (sheep and goats). However, many animal bones unearthed from the excavations are far from complete for various reasons; they are broken, missing, or burnt. This is why most of the bones cannot be identified to species with one hundred percent accuracy each time. This phenomenon is most common in sheep and goat bones due to their morphological and size similarities. This necessitates archaeozoologists to create a separate category for sheep/goat remains when entering data. During archaeozoological studies, after calculating the ratio of positively identified sheep remains to positively identified goat remains, new calculations applied to the artificially created sheep/goat column provide more accurate data on the percentage distribution of domestic animal species in herds [7,8,25,29,30,31,32,33,34,35].

Identifying any equine remains is a common challenge in archaeozoological research. Differentiating between horse, donkey, wild ass, and hybrid bone specimens at archaeological sites is a persistent challenge. Limited methodologies are available to distinguish between these types of equid bones [36]. Many of the current methods for identification are centered on the physical characteristics of teeth enamel and skull morphology, as evidenced by various studies [37,38,39,40,41,42].

**Figure 2:**
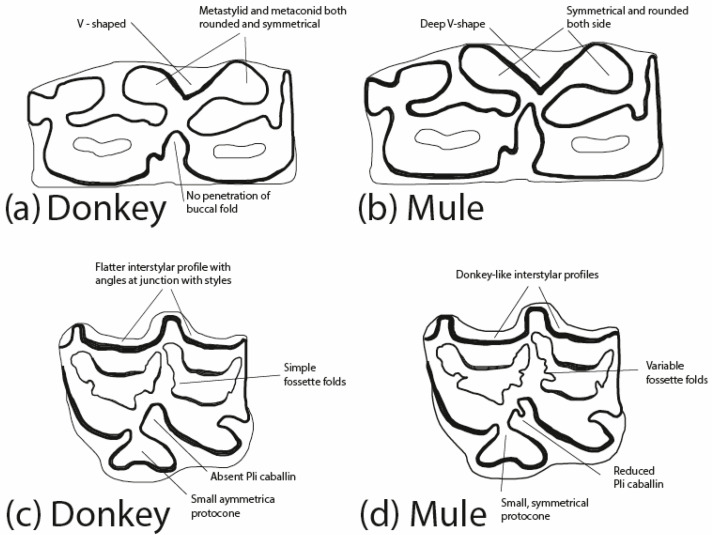
Detail of morphological differences between the lower donkey (**a**) and lower mule (**b**) teeth and the upper donkey (**c**) and upper mule (**d**) molars (re-drawn by A. Badem after Johnstone 2004, Figure 4.3).

As mentioned above, it is not easy to distinguish between the remains of different equid species. In this study, the excavated dental remains were used as a reference for the differentiation of species, and in particular, studies by Johnstone 2004 [43] (see Figure 2) and Mohaseb et al. 2023 [44] were used for the differentiation of equine dental remains. No ancient DNA (aDNA) or zooarchaeology by mass spectrometry (ZooMS) methods were used in this study; these techniques sometimes reveal problems in providing a trustworthy identification. In addition, it is doubtful that the bones from Derekutuğun would yield any useful results from these genomic and proteomic methods as a result of the oxidation formed on the bone remains. Bendrey 2007 [45] was also used to identify pathological marks on the remains.

## 3. Results

### 3.1. Summary of Livestock Economy and Meat Consumption in Derekutuğun*4*

The animal bone assemblage from Derekutuğun is predominantly composed of mammal remains, with only a small number of non-mammal remains, such as birds and turtles. Among the 4426 animal remains analyzed, only 11 non-mammal remains were detected. The majority of the identified mammal remains were from domestic animals, especially small ruminants such as sheep and goats. Other domestic animals identified included pigs, cattle, donkeys, mules, and dogs. On the other hand, wild animal remains were in the minority, with red deer and foxes being the dominant species. This composition reflects the animal-based economy of the site and provides insights into the animal husbandry practices during the Early Bronze Age at Derekutuğun (Table 2).

The livestock in Derekutuğun remained relatively stable over time, with sheep being the most commonly kept animals, followed by pigs and cattle (Table 2). The small ruminants were primarily used to meet the demand for meat among the inhabitants/miners. Sheep and goats were primarily raised between the ages of two and six, with the majority slaughtered at around two to three and a half years old. This indicates a pattern of slaughter primarily associated with the exploitation of animals for meat [33] (Diagram 6.9-18). Pigs were primarily bred for the consumption of meat, with most being slaughtered between the ages of ten months and two years [33] (Diagram 6.33-37). Cattle made up a smaller portion of the livestock and were primarily slaughtered to meet the demand for meat [33] (Diagram 6.24-28). There is an increase in the number of donkey and mule remains over time (Table 2).

Only 14 bone remains out of 4426 faunal remains were identified as dog bones, except in the Late EBA III Period. Insufficient epiphysis data are available within the faunal assemblage to make accurate estimations of age. Additionally, no evidence of human impact, such as fire or cut marks, was detected on the dog bones.

In terms of meat consumption, beef was the most consumed, followed by pork and mutton. The analysis shows that livestock played a significant role in meeting the demand for meat, with only a small percentage covered by wild animals (Table 2).

### 3.2. Donkey and Mule Remains from the Bone Assemblage of Derekutuğun

Remains of donkeys and mules account for approximately 1.8% of the identified mammal remains and represent about 4.7% of the total bone weight. No horse remains have been found in the materials that were studied. The presence of donkey and mule remains increased during the EBA III/Karum Period in Derekutuğun. There is a noticeable fluctuation in the weight of donkey and mule remains over time among the identified domestic animals. The fluctuation is approximately 3% in the Early EBA III Period, decreases to about 1.5% in the Late EBA III Period, and then sharply increases to 6.7% in the EBA III/Karum Period (Table 2). Table 3 shows the distribution of equid remains’ skeletal elements by stratigraphy.

Several specific dental remains have been identified and classified as either belonging to a donkey or a mule based on distinct morphological features. The high frequency of worn dental surfaces and fused joints suggests that the majority of the recorded remains are from adult animals.

**Figure 3:**
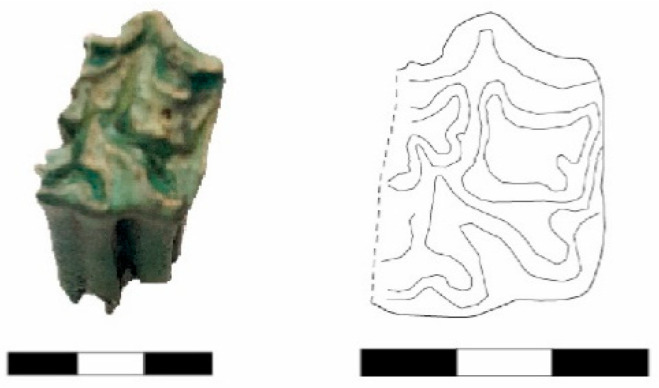
An upper molar from the Early EBA III Period, belonging to a donkey (Basket No. 70141).

A maxillary molar (Basket No. 70141) from the Early EBA III Period has been identified as belonging to a donkey. This identification is based on the typical features of the molar, including the absence of “*pli caballin*”, a simple fossette fold, and a small symmetrical protocone (Figure 3) (compare Johnstone 2004: Figure 4.3e and Table 4.1).

**Figure 4:**
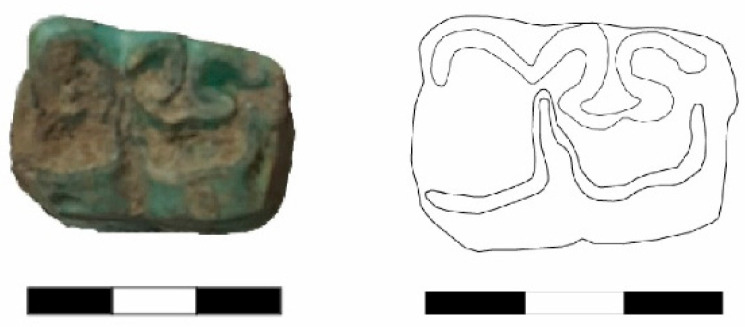
A lower jaw molar of a mule (Basket No. 8493) from the Late Early Bronze Age III Period.

A lower jaw molar (Basket No. 8493) from the Late EBA III Period is classified as a mule, particularly due to the deep penetration of the buccal fold into the so-called “*neck*” (Figure 4) (compare Johnstone 2004: Figure 4.3c and Table 4.1).

**Figure 5:**
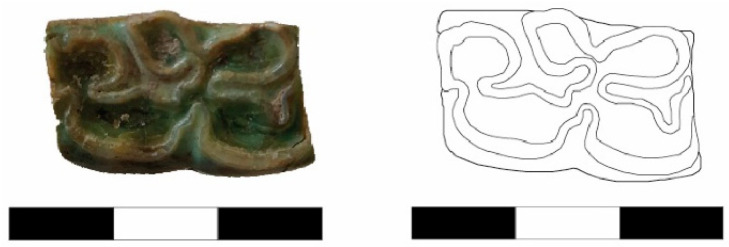
This is a lower jaw molar of a donkey (Basket No. 60130) from the EBA III/Karum Period.

A lower jaw molar (Basket No. 60130) from the EBA III/Karum Period has been identified as belonging to a donkey. This conclusion is based on features such as the absence of penetration of the buccal fold into the so-called “*neck*”, nearly symmetrical rounded double knots, and a shallow “V” shape lingual fold (Figure 5) (compare Johnstone 2004: Figure 4.3b and Table 4.1).

**Figure 6:**
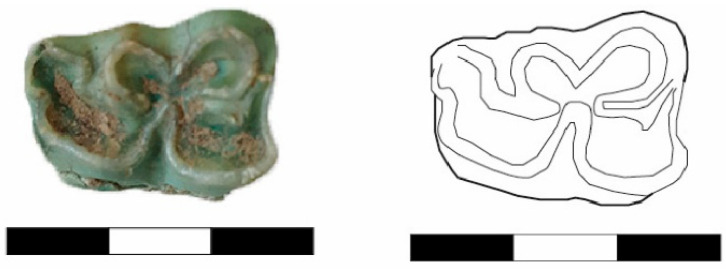
A lower jaw molar of a mule (Basket No. 60074) from the EBA III/Karum Period.

Once again, a lower jaw molar (Basket No. 60074) from the EBA III/Karum Period is classified as a mule due to its deep “V”-shaped lingual fold and the deep penetration of the buccal fold into the so-called “*neck*” (Figure 6) (compare Johnstone 2004: Figure 4.3c and Table 4.1).

**Figure 7:**
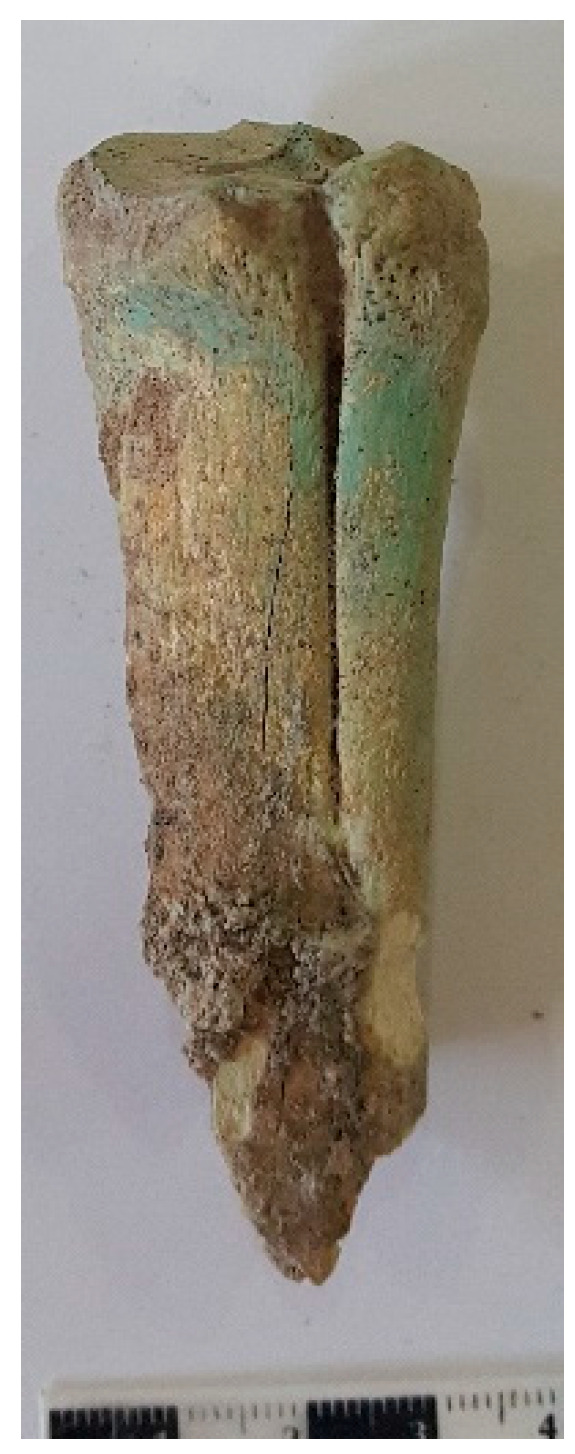
A pathological lesion was found on a mule’s metatarsal bone (Basket No. 60021) from the EBA III/Karum Period.

A pathological trace was found on the metatarsal (MT) remains of a possible mule (after its size) (Basket No. 60021) from the EBA III/Karum Period. In most cases, a pathological condition affecting the metapodia leads to the ossification of the ligament located between the metapodial shafts, known as *desmoiditis ossificans ligamentum interosseum* or “splints” [45] (p. 208) and [46]. The side MT is attached to the main MT (III) as a result of the individual’s hard work over time (Figure 7).

Splints, primarily involving the interosseous ligament between metacarpal bones, can cause periostitis, leading to the formation of new bone (exostoses). Factors contributing to this reaction include trauma, strain from excessive training, faulty conformation, imbalanced nutrition, or improper shoeing, particularly in immature horses [46,47].

Pathological evidence on the bones indicates that these animals were used for labor. Especially, traces on the metatarsal bones confirm that mules were used for strenuous tasks such as transporting, pulling, and possibly riding.

## 4. Discussion

Kill-off patterns indicate that the livestock in Derekutuğun were frequently slaughtered, as there was a reasonable correlation between fodder and body weight. After reaching a certain age, animals stop gaining weight despite consuming fodder, which makes it unprofitable to keep some animals in the herd due to the unprofitable correlation between fodder and body weight [7,15]. The young ruminants may have been killed to obtain milk from the females. However, there are no very solid data on the kill-off pattern to prove a massive usage of milk, presumably for household use in Derekutuğun. It is known that dairy products have been produced and consumed in the region since the Late Chalcolithic Period, after the studies of Bartosiewicz and Gllis [48] (pp. 78–79). Textile production should have also been a household activity, as evidenced by the small number of weaving instruments found in the archaeological contexts of Derekutuğun.

The remains of donkeys and mules from Derekutuğun may not make up a large portion of the identified mammalian remains. Nevertheless, they provide some of the earliest evidence of the existence of donkeys and, in particular, mules in Central Anatolia. The significant rise in the presence of donkeys and mules during the EBA III/Karum Period is recognizable. The increase in the number of donkeys and mules in the settlement can be attributed to the increased trade in copper, driven by a surge in demand from merchants (see also Table 1).

The remains of donkeys and mules from Derekutuğun have been identified, especially through dental remains. Two equid remains, including a P4 from a mandible and a cervical vertebra, were identified as belonging to a horse from the site of Yarıkkaya^5^. However, the researchers were unable to determine whether the bones belonged to a wild or domestic horse. Although the P4 was identified as a horse from Yarıkkaya, the nearly symmetrical and rounded double knot on both sides, as well as the absence of penetration of the buccal fold into the so-called “*neck*”, could indicate that this P4 might have belonged to a donkey (see Boessneck, J. and Wiedemann U. 1978, Abb. 16 compare with Johnstone 2004, Table 4.1. and Figure 4.3b).

A complete metatarsal of an equid was found from the mixed material of Büyükkale^6^. The Chalcolithic/EBA sample has been classified as belonging to a donkey based on the biometric results (A metatarsal from the Chalcolithic/EBA period has been classified as belonging to a donkey based on the biometric results). Since the existence of the donkey cannot be traced back to the Chalcolithic Period, this find should be dated to the Early Bronze Age [50] Table 27.

Arbuckle wrote about the equid remains from Acemhöyük as follows^7^. Domestic donkeys first appeared in Anatolia during the Late Chalcolithic Period, possibly due to the economic influence expansion from the northern Levant in the Uruk Period. The polity centered at Acemhöyük played a key role in the Assyrian trade network and internal Anatolian trade systems, relying on the use of donkeys as pack animals. The presence of donkey remains in Levels IV and V and two specimens in EBA Level XI suggest that Acemhöyük’s role as a significant trade node may have been present since the third millennium BC. Most equid remains are initially identified as either “large” or “small” equids, with the possibility of hybrids, mules, and hinnies within each group. Hemione (*Equus hemionus*) × donkey hybrids, which were documented in Bronze Age Mesopotamia, may have also been present in Anatolia [52]. However, no equid remains were certainly classified as a mule from Acemhöyük. Equid remains from Çamlıbel Tarlası have not been further identified^8^. Atici mentioned that he identified 14 donkey and 20 *Equus* sp. (equid) remains from the Lower Town of Kültepe- Kaneš, but the material is not detailed [53].

Sallaberger noted that texts from Mari (an ancient trade city in modern-day Syria) dating back to the third millennium BC mentioned specialists in breeding or suppliers of “*big donkeys*” [54] (p. 341). Barjamovic reported in the early second millennium texts of Kaneš about the donkey caravans containing “*black donkeys*” [55] (p. 88). Goulder listed the attributes of the black donkey after referencing the Kaneš text, describing them as large, strong, and young [56,57]. These types of donkeys were commonly used as pack animals and were not as costly as riding donkeys [56] (p. 258).

Dercksen writes about the donkeys after referencing the Old Assyrian written sources that the donkeys and their equipment were purchased in the city for trade. The merchandise and most of the surviving donkeys were sold for silver and gold in Anatolia. The goods were often exchanged for copper, which was widely used as a means of payment in the region [58] (p. 69). He adds as well that market forces influence supply and demand in the economies of Assur, Babylonia, and Anatolia, but their influence is not absolute. Increased production as a result of growing demand is observed in textiles in Babylonia and Assur, as well as in the breeding of donkeys and the manufacture of their harnesses [58] (p. 75).

Donkey breeding and the production of their harnesses in Mesopotamia constituted an important market and an indispensable part of the trade network. It is important to acknowledge the human employees who accompanied the caravans. Assyrian merchants profited from pack animals in two ways: first, they used them for their caravans to transport their exclusive goods in distant lands, and second, they sold or traded the animals upon reaching their final destinations in Anatolia to generate additional profits.

The earliest evidence of mules in Derekutuğun dates back to the Late EBA III Period. Michel writes about the equids after referencing the Old Assyrian (btw. 2025–1552 BC) tablets from Kaneš, where horses are not mentioned in the tablets. However, they do mention the trade of hybrids (mules and hinnies); crossbreeding between a donkey and a horse results in sterile offspring. Hybrids were well known in the Ancient Near East during the third millennium BC, as evidenced by both archaeological and textual records. The crossbreeding of asses and horses was undertaken to produce equids that were more robust than donkeys and more resistant than horses. The goal was to create a hybrid with desirable traits from both species. The Old Assyrian documentation mentions the word “*perdum*” as an equid hybrid, which has been translated as “*mule*” and appears frequently in the tablets, documenting its trade and use [59] (p. 192).

The references to the mule/perdum in the Kaneš archives indicate the high price of this animal in Anatolia. The cost of the mule/perdum was nearly four times the price of a donkey. Michel also documented the trade of mules in Anatolia, noting that the perdum trade was primarily conducted by Assyrian merchants, with minimal involvement from Anatolians. The Assyrian merchants acted as intermediaries, purchasing animals from local authorities in Burušhattum, Wahšušana, and Šaladuwar along the Tuz Gölü (translation, Salt Lake; a lake located in the center of Central Anatolia), and then selling them to local people in Kaneš and other Anatolian cities. The perdum trade often involved the commercialization of native iron, a scarce and expensive metal. Additionally, the trade of iron appeared to be under the control of local authorities [59] (p. 193).

The written records prove the existence of the mule/perdum and its trade in Anatolia. The interbreeding took place in Anatolia, likely carried out by the native population and traded by Assyrian merchants. Therefore, it was not surprising to find the remains of mules in Derekutuğun even before the Karum Period in Anatolia.

## 5. Conclusions

Archaeozoological data show that one of the earliest known donkeys and mules of Anatolia were kept in the settlement of Derekutuğun, and that these animals simplified people’s lives. Burden animals were kept at the settlement of Derekutuğun from the EBA III Period onwards, and their numbers increased significantly over time.

The miners utilized donkeys and mules to transport their heavy loads from the mines to the settlement for extracting the copper ore. After the extraction, the intermediate products were once again transported to the markets or wholesalers by the donkeys and mules. Derekutuğun was a relatively small community, but it specialized in the processing of copper ore. The density of mining and extraction activities as well as the existence of donkeys and mules made this settlement an important starting point for a trade network.

## Figures and Tables

**Table 1:** Stratigraphy of the Derekutuğun Miner Settlement.

Periods	Roman Period	Hellenistic Period	Iron Age	Hiatus	Karum (Assyrian T. Col. Ages)	Transition from EBA III to MBA	EBA III	EBA II	EBA I	Chalcolithic Period
**C-14** **Dating**	--	4th and 3rd c. BC.	--	--	2200–1900 BC.	--	--	--	--	--

**Table 2:** Number and weight distribution of the animal bone remains from different levels of Derekutuğun settlement (modified by the author after Sarı 2019, Table 5.8–5.11).

	**Number of the Studied Material (Number)**				**Weight of the Studied Material (Gram)**			
**Derekutuğun Periods**	**E-EBA III/N**	**L-EBA III/N**	**EBA III-Karum/N**	**Derekutuğun** **Total/N**	**E-EBA III/W**	**L-EBA III/W**	**EBA III-Karum/W**	**Derekutuğun Total/W**
Identified mammal remains total	573	754	1560	2888	11,291.1	11,462.4	26737.3	49,490.8
Unident. remains total	131	600	796	1527	1152.5	3516.5	3981.9	8650.9
**Mammal remains total**	**704**	**1354**	**2356**	**4415**	**12,443.6**	**14,978.9**	**30,719.2**	**58,141.7**
Mammal remains total	704	1354	2356	4415	12,443.6	14,978.9	30,719.2	58,141.7
Not mam. remains total	3	0	9	11	4,2	0	22.5	26.7
**Processed material TOTAL**	**707**	**1354**	**2365**	**4426**	**12,447.8**	**14,978.9**	**30741.7**	**58,168.4**
	**NISP (Number of the Identified Species in %)**				**WISP (Weight of the Identified Species in %)**			
**Derekutuğun/identified faunal remains—%**	**E-EBA III/N**	**L-EBA III/N**	**EBA III-Karum/N**	**Derekutuğun** **Total/N**	**E-EBA III/W**	**L-EBA III/W**	**EBA III-Karum/W**	**Derekutuğun Total/W**
Dog, *Canis familiaris*	0	0.8	0.51	0.48	0	0.23	0.28	0.21
Sheep, *Ovis aries*	38.4	37.54	32.62	35.32	21.03	24.22	12.39	17.07
Goat, *Capra hircus*	3.2	10.35	11.53	9.28	1.66	9.5	6.34	6.03
Pig, *Sus domesticus*	38.4	33.43	26.75	30.8	33.68	31.31	27.31	29.69
Cattle, *Bos taurus*	16.9	15.9	23.05	19.94	39.01	31.95	44.86	40.54
Donkey and Mule, *Eq. asinus & Eq. mulus*	1.08	0.92	2.61	1.86	3.03	1.46	6.74	4.67
**Domestic mammals total—%**	**97.98**	**98.94**	**97.07**	**97.68**	**98.41**	**98.67**	**97.92**	**98.21**
Wild Boar or Pig	0	0	0.13	0.08	0	0	0.15	0.08
Large cervid or bovid	0.35	0.53	0.64	0.55	0.61	0.79	0.45	0.57
**Wild/Domestic mammals total—%**	**0.35**	**0.53**	**0.77**	**0.63**	**0.61**	**0.79**	**0.6**	**0.65**
Hare, *Lepus capensis/europaeus*	0	0	0.13	0.08	0	0	0.02	0.01
Fox, *Vulpes vulpes*	0.52	0.13	0.38	0.34	0.23	0.22	0.11	0.16
Wolf, *Canis lupus*	0	0	0.13	0.08	0	0	0.21	0.11
Carnivora unident.—middle sized	0.2	0	0	0.03	0.18	0	0	0.04
Roe deer, *Capreolus capreolus*	0.35	0	0.06	0.1	0.28	0	0.02	0.08
Fallow deer, *Dama dama*	0.2	0.13	0.06	0.1	0.12	0.12	0.09	0.1
Red deer, *Cervus elaphus*	0	0	0.64	0.34	0	0	0.8	0.43
Cervidae indet.	0	0.27	0.19	0.21	0.13	0.2	0.15	0.15
**Wild mammals total—%**	**1.27**	**0.53**	**1.59**	**1.28**	**0.94**	**0.54**	**1.4**	**1.08**
Aves	0.2	0	0	0.03	0.01	0	0	0.01
Testudinae	0.2	0	0.57	0.38	0.03	0	0.08	0.05
**Not mammal remains—%**	**0.4**	**0**	**0.57**	**0.41**	**0.04**	**0**	**0.08**	**0.06**
**Identified material TOTAL—%**	**100**	**100**	**100**	**100**	**100**	**100**	**100**	**100**
**Derekutuğun/unident. mammal remains total—%**	**E-EBA III/N**	**L-EBA III/N**	**EBA III—Karum/N**	**Derekutuğun** **Total/N**	**E-EBA III/W**	**L-EBA III/W**	**EBA III—Karum/W**	**Derekutuğun Total/W**
Unident., medium	48.86	61.67	69.47	64.63	23.51	29.26	35.12	31.19
Unident., med. to large	44.27	31	15.71	24.17	57.61	50.75	24.75	39.7
Unident., large	6.87	7.33	14.82	11.2	18.88	19.99	40.13	29.11
**Unident. mammal remains total—%**	**100**	**100**	**100**	**100**	**100**	**100**	**100**	**100**

**Table 3:** The table shows the distribution of equid skeletal elements by stratigraphic level.

Equid Remains	Early EBA III	Late EBA III	EBA III/Karum	Late EBA III/MBA ?	Total
**Dental**					
Maxilla	1		4		**5**
Mandible	1	2	11		**14**
Max. or Man.		1			**1**
**Bones**					
Scapula			1		**1**
Humerus			7	1	**8**
Radius			5		**5**
Ulna			3		**3**
Radius with ulna			1		**1**
Metapodium	1	2	3		**6**
Pelvis			1		**1**
Femur	1		3		**4**
Tibia	1		1		**2**
Phalanges		1	1		**2**
**Total**	**5**	**6**	**41**	**1**	**53**

## Data Availability

Data are contained within the article.

## Notes

1 See also Gündem, C.Y. Derekutuğun Yerleşimi Arkeozoolojik Çalışmaları Işığında Hayvan Kalıntılarının Tartılmasının Önemi. *Antropoloji* **2019**, *38*, 118–125. https://doi.org/10.33613/antropolojidergisi.630376.

2 S. Sarı and E. Emlük assisted the author in identifying the faunal remains, while E. Gündem recorded the identified material.

3 The Thesis Is Available to the Public at the National Thesis Center. Available online: https://tez.yok.gov.tr/UlusalTezMerkezi/giris.jsp (accessed on 30 April 2024).

4 Some of the numerical data in this section was taken from S. Sarı’s thesis, and the author added his comments. The author of this study is also working on a chapter titled “Miners’ Menu vs. Farmers’ Menu on the Highlands of Northeast of Central Anatolia”. As the title of the chapter implies, it focuses on the relationship between communities and their key animal species in various geographical locations and across different business sectors. He also discusses the connection between the settlement patterns in the region and the preferred capital animals, as well as their locations.

5 Yarıkkaya is located in Çorum/Türkiye, a hillside settlement that was established during the early periods of the Early Bronze Age (EBA). Yarıkkaya is located approximately 4 km north of Büyükkaya and very close to Çamlıbel Tarlası as well. Boessneck and Wiedemann identified approximately 500 animal bone remains from the site [49].

6 The animal remains from Büyükkale were studied by von den Driesch and Pöllath. The site is located in Çorum, which later became known as the capital city of the Hittites/Boğazköy. The study utilized data from the Early Chalcolithic and Early Bronze Age [50].

7 It was determined that at least 12 layers were associated with the Early Bronze and Assyrian Trade Colonies Ages in the Acemhöyük Mound. The Lower City was inhabited only during the Assyrian Trade Colonies Age. While the city gradually developed from the Old Bronze II (2500 BC) period, it experienced its most prosperous period during the Age of Assyrian Trade Colonies [51].

8 Çamlıbel Tarlası is a Late Chalcolithic site located near Büyükkale. This rural settlement was occupied for approximately 120 years (from around 3590 to 3470 BC) and is situated on a small plateau in a mountainous area. The animal bone remains were identified by Bartosiewicz. Bartosiewicz and Gllis discuss the animal-based economy in Çamlıbel Tarlası during the Late Chalcolithic Period. The data show that cattle have a longer lifespan compared to pigs, which are slaughtered at younger ages, while the age distributions for small ruminants fall between these two extremes. Pigs were primarily raised for meat, while cattle may have been used for draft power and dairy products. It is also likely that sheep and goats were milked, and wool production was considered with sheep [48] (pp. 78–79).
